# Educational degree differences in the association between work stress and depression among Chinese healthcare workers: Job satisfaction and sleep quality as the mediators

**DOI:** 10.3389/fpubh.2023.1138380

**Published:** 2023-03-30

**Authors:** Afei Qin, Fangfang Hu, Wenzhe Qin, Yaru Dong, Menghua Li, Lingzhong Xu

**Affiliations:** ^1^Centre for Health Management and Policy Research, School of Public Health, Cheeloo College of Medicine, Shandong University, Jinan, Shandong, China; ^2^National Health Commission (NHC) Key Laboratory of Health Economics and Policy Research (Shandong University), Jinan, Shandong, China; ^3^Center for Health Economics Experiment and Public Policy Research, Shandong University, Jinan, Shandong, China

**Keywords:** work stress, depression, job satisfaction, sleep quality, healthcare workers, educational degree

## Abstract

**Background:**

Depressive status of medical personnel worldwide and especially in China is an important public health and social problem. There is a strong relationship between education and depression, but no studies have studied grouping healthcare workers (HCWs) with different educational degree to discuss whether there are differences in the factors that affect depression. This study aims to examine the role of job satisfaction and sleep quality in the relationship between work stress and depression among Chinese HCWs, and teste whether the mediation models are differed by the differences of educational degree.

**Methods:**

Patient Health Questionnaire-9 (PHQ-9) scale was used to test depression. Work stress was assessed using the Challenge-blocking stress scale (CBSS). Sleep quality was assessed using the Pittsburgh Sleep Quality Index (PSQI). HCWs’ satisfaction with their current work was assessed using the Job Satisfaction Index (JSI). The representative sample of HCWs was chosen using a multi-stage stratified cluster random sampling procedure and 844 HCWs were utilized to the statistical analysis of the study.

**Results:**

In the overall sample, sleep quality could mediate the relationship between work stress and depression in healthcare workers (*p* < 0.001, CMIN/DF = 3.816, GFI = 0.911, AGFI = 0.886, IFI = 0.943, TLI = 0.933, CFI = 0.942, RMSEA = 0.058, SRMR = 0.055, AIC = 1039.144), and the mediating effect accounted for 36.5%. After grouping educational qualifications, the model with sleep quality and job satisfaction as mediating variables reported a better fit in the group with low educational qualifications. The intermediary effect accounted for 50.6 and 4.43%, respectively. The highly educated group only has sleep quality as an intermediary variable in the structural model, and the mediating effect accounted for 75.4% (*p* < 0.001, CMIN/DF = 2.596, GFI = 0.887, AGFI = 0.857, IFI = 0.937, TLI = 0.926, CFI = 0.937, RMSEA = 0.044, SRMR = 0.056, AIC = 1481.322).

**Conclusion:**

In the overall sample, sleep quality could mediate the relationship between work stress and depression in HCWs. Among HCWs with technical secondary school education and below, job satisfaction can mediate the positive relationship between work stress and depression, while this mediating effect is not significant among HCWs with college degree and above.

## 1. Introduction

As the world’s most populous country, by the end of 2021, China had a total of nearly 14 million healthcare workers (HCWs) (1). One out of every 101 persons works in healthcare. HCWs are experiencing unprecedented workloads and challenges as demand for health care rises. Especially in recent years, the COVID-19 epidemic has devastated the world, putting significant strain on HCWs worldwide. HCWs represent the defense front lines who take care of patients every time a pandemic or an epidemic arises (2), and they operate under more intense strain than other employees. Prolonged exposure to work stress can result in depression in HCWs (3). Many studies have reported a high rate of depression among HCWs (4, 5). Among HCWs, depression is a prevalent mental illness with nuanced causes. A rate 2–3 times greater than the general population, over 30% of resident doctors and 20% of hospital-based nurses experience depression (6, 7). Factors that contribute to depression include work stress, chronic illness, the doctor-patient interaction, workload, job satisfaction, sleep quality, and loneliness (8–10). HCWs were predisposed to depression as a result of specific occupational traits. The depressive state of medical workers globally, particularly in China, is a major public health and social issue. We must now discover which factors may be beneficial in determining the most effective ways to enhance this phenomenon.

Chinese HCWs have generally experienced psychiatric emotional illnesses including depression. Depression, in addition to producing physiological problems, can also lead to serious psychological problems, such as suicide (11, 12). According to studies, anxiety/depression was a factor in a total of 46 instances (45 females) of nurse suicides in China between 2007 and 2016 as well as 51 cases (16 females) of doctor suicides in China between 2008 and 2016 (11, 13). Of course, male doctors are no exception, and even more serious (13, 14). Nevertheless, due to the complexity of depression’s pathophysiology and the paucity of effective treatments, there were few effective therapies (15). Additionally, there were numerous complicated elements that might contribute to depression, including cultural, psychological, and biological ones (15). In order to identify effective depression intervention solutions, it is imperative to continue researching the pertinent elements of depression.

Numerous researchers have found a link between job stress and depression, whether they used HCWs as their research subjects or other volunteers (8, 16). There were few studies, nevertheless, have been conducted to investigate the likelihood of a mediation association between occupational stress and depression among HCWs. The research has revealed a link between job satisfaction and depression among HCWs (17), as well as a link between work stress and job satisfaction (18). Among HCWs, studies have revealed a significant mediating role between mood exhaustion and social support between work stress and depression (19), as well as the effects of job satisfaction and depression on willingness to leave (20). Quick et al. emphasized human psychology and mentality, which is the theoretical model of response theory, and hold that job stress is a reaction-based paradigm (21). This connected mental and mental health to stress at work. According to Robbins’ model of stress theory (22), being under stress causes anxiety, depression, and decreased work satisfaction, which ties stress and job satisfaction together. Sleep issues frequently manifest before a new or repeated episode of severe depressive illness, according to research by Peter et al. (23). To summarize, it is essential to investigate the impact of job satisfaction and sleep quality in mediating the relationship between work stress and depression. There were indications that the association between work stress and depression may be influenced by job satisfaction and sleep quality. However, few studies have investigated the mediating role of job satisfaction between job stress and depression.

Poor mental health disorders like depression are frequently accompanied by poor sleep quality. Sleep problems are widespread illnesses that are very curable. The onset of a variety of adult mental problems is associated with sleep abnormalities (24), such as depression and anxiety. Numerous research, including those involving senior citizens and dentistry students, have confirmed the strong link between poor sleep and depression (25, 26). This connection has also been verified in HCWs (27). As nurses in many departments of health institutions have confirmed, sleep disorders were not only directly related to job stress but also inexorably connected to depression (28). However, it has not been researched, especially among this specific group of HCWs, if poor sleep quality can mediate the link between job stress and depression.

It is public knowledge that HCWs are generally more educated than other occupations. By the end of 2020, 15.1% of all the population in China had college degree or above, and 80.5% of all HCWs had college degree or above (29, 30). HCWs with college degree or above accounted for 5.3 times of the total population. Depression and educational attainment have been shown to interact in pregnant women (31). In South Korea’s population aged 45 and older, education influenced depression through different underlying mechanisms, most notably through the development of cognitive abilities (32). A study found that among Chinese nurses, the degree of education had a statistically significant effect on depression (33). Community mental health care workers who were younger than 30 years, single, and received higher education showed higher depressive symptoms, and higher level of education may bring them more psychiatric issues during COVID-19 epidemic (34). Altogether, there was sufficient evidence to conclude that education was substantially connected with depression overall, and especially among HCWs.

In accordance with the content afore mentioned, there were insufficient presents the findings at how job satisfaction and sleep quality affected the association between depression and work stress, especially among HCWs. There is a significant association between education and depression, and numerous researchers have found that those with higher levels of education were more likely to experience depression. However, no studies have compared groups of HCWs with various levels of education to determine whether there are variations in the factors that influence depression. Therefore, the following hypotheses are proposed in this study: (1) among Chinese HCWs, job satisfaction and sleep quality mediated the link between work stress and depression; (2) the mediation models were differed by the differences of educational degree. In the context of China’s massive medical workforce and its increasingly severe depressive symptoms, identification of the factors that contribute to depression among HCWs and the mediating mechanisms can encourage administrators of hospitals and other medical institutions to take proactive and successful actions to reduce risk factors and strengthen protective factors. The management of medical institutions can develop efficient strategies to fulfill the various demands of medical staff members according to their varied educational backgrounds thanks to the disparities brought about by academic degrees.

## 2. Materials and methods

### 2.1. Participants

Data were gathered from the 2020 Household Health Interview Survey, which attempted to investigate the practice status and work status of doctors in primary medical institutions. It included information on basic personal information, work and assessment, job satisfaction, work stress, burnout, sleep quality and depression. The representative sample of HCWs was chosen using a multi-stage stratified cluster random sampling procedure, which combined multiple methodologies often employed in large-scale epidemiological sample surveys in order to get sampling results that are more representative of the real population. On-the-spot investigation was conducted in six counties (districts) of Tai’an City, Shandong Province, China. The investigating body comprised the corresponding village’s community health service center, township health center, and its subordinate community health service station, or village clinic. First, three to four townships were randomly selected from each district (county) of Tai’an City using a probability sampling method proportional to the scale, according to the local economic development level and geographical position. Second, a total of 160 villages (communities) were randomly selected from each township (communities). Third, health providers were recruited from selected primary health facilities in each village (community). All general practitioners, village physicians, and other medical staff who were on duty or at the relevant institution on the same day were included as participants, as were those who were aware of the survey’s purpose, gave their agreement willingly, and engaged in it. Individuals who found it difficult or impossible to communicate as well as those who refused to participate in the survey were excluded. We obtained informed consent from the respondents after thoroughly outlining the rationale for gathering their personal data and how it would be used. Each respondent was interviewed face-to-face by in-depth trained investigators for no less than 30 min.

The sample size was determined as follows:


$$N=\frac{\mu_{\alpha }^{2}\times p\left(1-p\right)}{\sigma^{2}}\times deff$$

The confidence level was 95%, $\mu_{\alpha }=1.96$, the relative error was 5%, σ=5%×75%, and deff=1.5. N = 768 was calculated. Decided on an invalid questionnaire rejection rate of 10% and a minimum sample size of 845. There were 860 questionnaires distributed in all, and 849 of them were returned, yielding a recovery rate of 98.72%. Five surveys with plenty of missing values were discarded. The survey data of the last 844 HCWs were utilized to the statistical analysis of the study. This project was approved by the Ethical Committee of Shandong University’s Centre for Health Management and Policy Research (approval number: LL20191220).

### 2.2. Measures

#### 2.2.1. Depression

The PHQ-9 (Patient Health Questionnaire-9) scale was applied in this research to assess depression. The PHQ-9 is a self-report screening instrument for depression symptoms containing nine items (3 points each) (35). The items of the PHQ-9 map onto the DSM-IV major depression criteria. The severity of the depression was indicated by the scale’s higher score. Based on past validation studies in cohorts, a total score of 10 or higher was deemed predictive of depression (36). The PHQ-9 was a valid and reliable indicator of the severity of depression (35, 37, 38). The Cronbach’s alpha coefficient of PHQ-9 scale in this study was 0.930, the Kaiser-Meyer-Olkin (KMO) value was 0.938, and the significance of the Bartley sphericity test was *p* < 0.001, which indicated good reliability and validity of PHQ-9.

#### 2.2.2. Work stress

The Challenge-blocking stress scale (CBSS) developed by Professor Cavanaugh (39) was utilized in this study to assess work stress. The self-rating scale has 11 items in two categories, six of which are challenging stressors and five of which are obstructive stressors. Challenging stress comprised 6 topics, such as “TIME urgency I experience”; There were five categories of obstructive stress, such as “Inability to clearly understand your work standards.” Likert 5 subscale was used in the questionnaire, with 1 ~ 5 representing “no pressure,” “some pressure,” “uncertain,” “relatively pressure,” and “very pressure” respectively. The participants had to assess how much stress had been brought on by the stressor over the previous 3 months considering their actual work environment. A higher score suggested that HCWs were under more stress. In this study, the demanding pressure scale’s Kronbach’s alpha coefficient was 0.901, with the challenging pressure’s alpha coefficient being 0.923 and the blocking pressure’s alpha coefficient being 0.875. The KMO value was 0.918, and the significance of the Bartley sphericity test was *p* < 0.001. The above results indicated good reliability and validity of the scale.

#### 2.2.3. Sleep quality

The Pittsburgh Sleep Quality Index (PSQI), which measures subjective sleep quality throughout the preceding 1-month period, was used to measure the quality of sleep in this study. PSQI was compiled by Dr. Buysse, a psychiatrist at the University of Pittsburgh in 1989 (40). This scale is suitable for the evaluation of sleep quality of patients with sleep disorders and mental disorders, and it is also suitable for the assessment of sleep quality of ordinary people. The PSQI consists of 19 self-rated questions. The 19 self-rated questions evaluate a wide range of sleep-related parameters, such as estimates of sleep duration and latency as well as the frequency and seriousness of sleep-related issues. Seven component scores, each weighted equally on a 0 ~ 3 scale, were created from these 19 elements. The global PSQI score, which ranges from 0 to 21, is then calculated by adding the seven component scores; higher numbers denote poorer sleep quality (40). Numerous investigations have utilized this scale, which has been found to have high reliability and validity (41). In this study, the Kronbach α coefficient of PSQI was 0.757, the KMO value was 0.741, and the significance of the Bartlett sphericity test was *p* < 0.001, which indicated good reliability and validity of PSQI.

#### 2.2.4. Job satisfaction

HCWs’ satisfaction with their current work was assessed using the tool developed by Schriesheim & Tsui (42), the Job Satisfaction Index (JSI). This instrument consists of a six-item questionnaire that is used to gauge how satisfied HCWs are with their current jobs. This test includes components that are identical to the aspects of job satisfaction: the nature of the work, supervision, coworkers, compensation, and promotion chances. A Likert scale of one (strongly disagree) to five (strongly agree) is included in the instrument. Higher scores indicate higher job satisfaction. Based on the previous investigation, the JSI scale was determined to be reliable with a Cronbach’s alpha of 0.95 (42). The Cronbach’s alpha for the current study was 0.893, the KMO value was 0.843, and the significance of the Bartlett sphericity test was *p* < 0.001, which indicated good reliability and validity of JSI.

#### 2.2.5. Covariables

Ten covariates, including sociodemographic variables, were included in the statistical analysis. The participants’ educational backgrounds were divided into two groups, technical secondary school and below and college and above. Gender was measured as male (0) and female (1). The participants’ ages were calculated using their date of birth and divided into three groups, ≤44 years old belong to young people (0), ≥45 years old belonging to middle-aged and older adults (1). District was assessed as urban (0) and rural (1) areas. Individual annual income was assessed as less than RMB 30,000 (0) and RMB 30,000 or more (1). The length of medical service of HCWs was divided into less than 20 years (0) and more than or equal to 20 years (1). The number of hours worked per day by participants was divided into two categories, less than 10 h (0) and more than or equal 10 h (1). Whether or not HCWs have participated in professional work training was evaluated by no (0) and yes (1). Participants with or without commercial health insurance were assessed as no (0) and yes (1).

### 2.3. Statistical methods

All data analysis procedures for this investigation were carried out using SPSS, version 27.0. First, descriptive analysis was used to examine the mean and standard deviation of continuous variables and the number and proportion of categorical variables. One-way ANOVA and Chi-square analysis were conducted to assess mean differences for variables across the educational qualifications. Second, one-way ANOVA was used to assess whether different categories of all covariates were different for depression. In this step, the overall situation, and the classification of educational degree were analyzed separately. Third, multiple regression was used to assess whether all covariates and continuous variables were statistically significant for depression. Similarly, in this step, the analysis of the overall situation and the classification of educational degree were made, respectively. To assess the average differences between various educational degrees, one-way ANOVA and chi-square analysis were utilized. Finally, Structural equation modeling (SEM) was employed *via* maximum likelihood using SPSS AMOS (version 26.0) to test the research model based on the data collected from HCWs in Tai’an City, Shandong Province, China.

There are several metrics that determine the degree of agreement between the hypothetical model and the observed data in structural equations. Typically, the root-mean-square error of approximation (RMSEA) of <0.08 means that the model is acceptable (43). In a model with a good fit, it is best to meet the following criteria: (1) the Goodness-of-Fit Index (GFI) should be greater than or equal to 0.90; (2) the Comparative Fit Index (CFI) should be greater than or equal to 0.90; (3) the Standardized Root Mean Square Residual (SRMR) should be less than or equal to 0.10; (4) Tucker Lewis Index (TLI) should be greater than 0.90 (44); (5) the Incremental Fit Index (IFI) should be greater than 0.90 (45); (6) the Adjusted Goodness-of-Fit Index (AGFI) should be greater than or equal to 0.80 (46). If the GFI does not reach 0.90 but is very close to 0.90, the fit of the structural model can still be considered acceptable if other indices are satisfied. Studies have decided this way (47).

## 3. Results

### 3.1. One-way ANOVA and Chi-square analysis of variables related to educational qualifications

The data from 844 HCWs in Tai’an, Shandong Province, China were included in the statistical analysis of this study. Among them, male participants accounted for 53.2%; 56.8% of participants were younger than 44 years old; the proportion of HCWs whose household registration was urban was 41.4%; the proportion of HCWs with an annual income of less than 30,000 yuan was 52.7%; the proportion of participants with chronic diseases was much smaller than that of those who do not have chronic diseases, accounting for less than a quarter (19.1% vs. 80.9%), and so on. More detailed data as well as other categorical variables are given in Table 1. Table 1 expounds that all 9 covariates were statistically significant for academic qualifications (*p* < 0.001 or *p* < 0.05). Participants scored a mean score of 35.20 on work stress (standard deviation = 7.97); The average score for job satisfaction was 18.85 (standard deviation = 5.48); The average score for sleep quality was 6.23 (standard deviation = 3.41); The mean score for depression was 5.34 (standard deviation = 4.72). Except for depression, which was significantly different in educational qualifications (*p* < 0.05), work stress, job satisfaction, and sleep quality were not statistically significant on educational qualifications (*p* ≥ 0.05).

**Table 1:** Description and univariate analysis among different educational degree.

Variables	Overall	Technical secondary school or below	College degree or above	*F*/*χ*^2^
Gender				61.046^a^^,^***
Male	449 (53.2)	279 (62.1)	170 (37.9)	
Female	395 (46.8)	139 (35.2)	256 (64.8)	
Age				36.089^a^^,^***
<44	479 (56.8)	194 (40.5)	285 (59.5)	
≥45	365 (43.2)	224 (61.4)	141 (38.6)	
Resident				147.404^a^^,^***
Urban	349 (41.4)	86 (24.6)	263 (75.4)	
Rural	495 (58.6)	332 (67.1)	163 (32.9)	
Income				76.939^a^^,^***
<30,000	445 (52.7)	284 (63.8)	161 (36.2)	
≥30,000	399 (47.3)	134 (33.6)	265 (66.4)	
Chronic				19.596^a^^,^***
Yes	161 (19.1)	105 (65.2)	56 (34.8)	
No	683 (80.9)	313 (45.8)	370 (54.2)	
Medical working years				27.218^a^^,^***
<20	310 (36.7)	117 (37.7)	193 (62.3)	
≥20	534 (63.3)	301 (56.4)	233 (43.6)	
Daily working hours				66.720^a^^,^***
≤10 h	390 (46.2)	134 (34.4)	256 (65.6)	
>10 h	454 (53.8)	284 (62.2)	170 (37.4)	
Attend training				13.278^a^^,^***
No	99 (11.7)	32 (32.3)	67 (67.7)	
Yes	745 (88.3)	386 (51.8)	359 (48.2)	
Commercial insurance				4.227^a^^,^*
No	609 (72.2)	315 (51.7)	294 (48.3)	
Yes	235 (27.8)	103 (43.8)	132 (56.2)	
CBSS	35.20 ± 7.97	35.68 ± 8.26	34.73 ± 7.65	3.016
JSI	18.85 ± 5.48	18.74 ± 5.60	18.96 ± 5.37	0.335
PSQI	6.23 ± 3.41	6.03 ± 3.47	6.42 ± 3.34	2.855
PHQ	5.34 ± 4.72	4.96 ± 4.79	5.70 ± 4.63	5.182*

CBSS, Challenge-blocking stress scale; JSI, Job Satisfaction Index; PSQI, Pittsburgh sleep quality index; PHQ, Patient Health Questionnaire-9.

a Expected count of 0 cells is less than 5.

* *p* < 0.05;

*** *p* < 0.001.

### 3.2. One-way ANOVA of covariables related to depression

When the academic qualifications of HCWs were not classified, the only covariate associated with depression was whether they had chronic diseases (*F* = 2.198, *p* = 0.001). When classifying the educational qualifications of medical personnel, the covariate associated with depression in the relatively low educational group was whether they had attended business training or not (*F* = 1.882, *p* = 0.011). In the relatively highly-educated group, the covariate associated with depression was whether interviewees have chronic diseases or not (*F* = 2.939, *p* < 0.001).

### 3.3. Multiple regression analysis of variables related to depression

When participants were not grouped by academic qualification, variables associated with depression were age (*B* = −0.688, *p* = 0.029), household registration (*B* = −0.952, *p* = 0.001), annual personal income (*B* = −0.57, *p* = 0.029), work stress (*B* = 0.130, *p* < 0.001), job satisfaction (*B* = −0.049, *p* = 0.029), and sleep quality (*B* = 0.765, *p* < 0.001). After grouping HCWs’ educational qualifications, age in either group was not statistically significant with depression (*p* > 0.05). In the group with relatively low educational qualifications, depression was associated with personal annual income (*B* = −0.828, *p* = 0.031), job stress (*B* = 0.154, *p* < 0.001), job satisfaction (*B* = -0.089, *p* = 0.006), and sleep quality (*B* = 0.689, *p* < 0.001). In the group with relatively high educational qualifications, depression was associated with household registration (*B* = −1.273, *p* = 0.001), chronic disease (*B* = −1.573, *p* = 0.002), work stress (*B* = 0.104, *p* < 0.001) and sleep quality (*B* = 0.834, *p* < 0.001). More detailed data analysis results are shown in Table 2.

**Table 2:** Multiple regression analysis of variables related to depression.

Variables	Total				Educational degree^a^				Educational degree^b^			
	*B*	*p*	95% CI		*B*	*p*	95% CI		*B*	*p*	95% CI	
			LB	UB			LB	UB			LB	UB
Gender	0.41	0.114	−0.098	0.918	0.704	0.068	−0.053	1.461	0.066	0.85	−0.624	0.757
Age	−0.668	**0.029**	−1.267	−0.069	−0.608	0.174	−1.485	0.269	−0.457	0.274	−1.279	0.364
Resident	−0.952	**0.001**	−1.494	−0.41	−0.513	0.252	−1.392	0.366	−1.273	**0.001**	−2.018	−0.529
Income	−0.57	**0.029**	−1.083	−0.058	−0.828	**0.031**	−1.582	−0.074	−0.502	0.166	−1.213	0.21
Chronic	−0.46	0.141	−1.072	0.152	0.276	0.498	−0.525	1.078	−1.573	**0.002**	−2.546	−0.601
Medical working years	0.305	0.33	−0.31	0.92	−0.284	0.566	−1.255	0.687	0.544	0.173	−0.239	1.328
Daily working hours	−0.261	0.306	−0.76	0.239	−0.17	0.659	−0.927	0.587	−0.346	0.312	−1.017	0.326
Attend training	0.015	0.968	−0.732	0.763	−1.246	0.061	−2.55	0.059	0.567	0.21	−0.321	1.455
Commercial insurance	−0.254	0.344	−0.779	0.272	−0.626	0.129	−1.433	0.182	−0.01	0.977	−0.689	0.669
Work stress	0.130	**<0.001**	0.097	0.164	0.154	**<0.001**	0.106	0.201	0.104	**<0.001**	0.057	0.151
Job satisfaction	−0.049	**0.029**	−0.094	−0.005	−0.089	**0.006**	−0.152	−0.026	−0.011	0.739	−0.073	0.052
Sleep quality	0.765	**<0.001**	0.69	0.84	0.689	**<0.001**	0.58	0.797	0.834	**<0.001**	0.732	0.936

Bold values are denotes statistically significant. CI denotes to confidence interval, LB denotes to lower bound, UB denotes to upper bound.

a Denotes to technical secondary school or below.

b Denotes to college degree or above.

### 3.4. The mediating role of job satisfaction and sleep quality in the relationship between work stress and depression

In this step, the mediation model was established for all participants’ data samples (recorded as model 1), and then the mediation model is constructed after the academic qualifications were classified (recorded as model 2).

**Figure 1:**
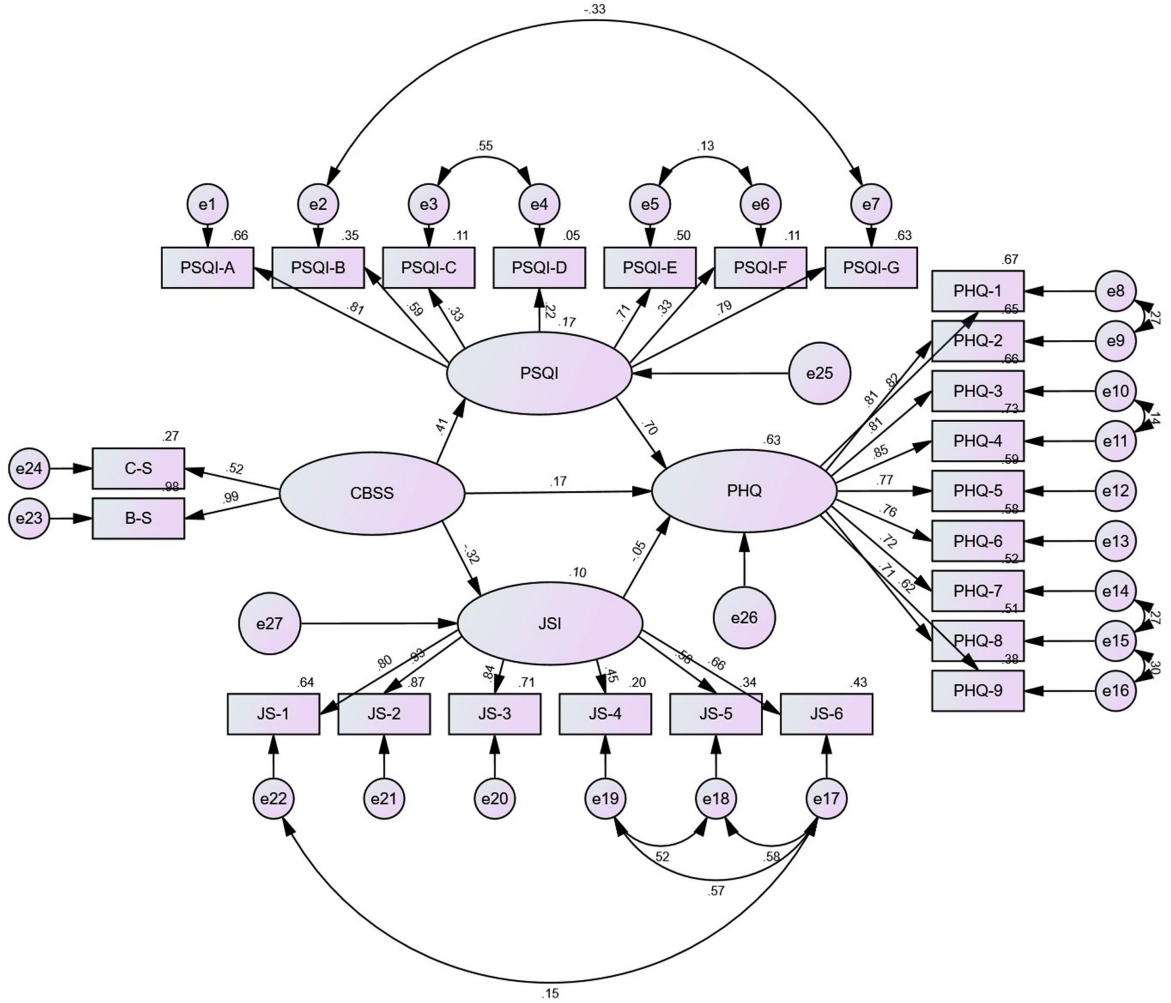
Results of the SEM analysis of the effects of sleep quality and job satisfaction mediate the association between work stress and depression among the whole sample. All the coefficients in the figure are standardized and significant at 0.001 level. Except that the path coefficient of JSI to PHQ is insignificant (*p* ≥ 0.05).

Work stress and sleep quality were statistically significant for depression when HCWs’ educational qualifications were not classified, while job satisfaction was not statistically significant for depression (*B* = −0.045, *p* = 0.123). Sleep quality had a significant incomplete mediating effect between work stress and depression, and the mediating effect accounted for 36.5%. However, job satisfaction did not mediate the relationship between work stress and depression, for job satisfaction was not significant for depression. The results about their relationship and 95% confidence intervals are all presented in Table 3. The model diagram of Model 1 is shown in Figure 1. Model 1 reported good fit of the mediating effect model, as detailed in Table 4.

**Table 3:** Standardized effects and 95% CI.

Grouping situation	Paths	Beta coefficient	*p*	95%CI	
				Lower	Upper
Total	Work stress → Sleep quality	0.413	0.012	0.337	0.493
	Sleep quality → Depression	0.703	0.007	0.659	0.759
	Work stress → Job satisfaction	−0.319	0.006	−0.401	−0.225
	Job satisfaction → Depression	−0.045	0.123	−0.094	0.000
	Work stress → Depression	0.167	0.006	0.109	0.234
Educational Degree^a^	Work stress → Sleep quality	0.465	0,01	0.300	0.604
	Sleep quality → Depression	0.598	0.007	0.497	0.693
	Work stress → Job satisfaction	−0.251	0.009	−0.351	−0.115
	Job satisfaction → Depression	−0.097	0.022	−0.164	−0.026
	Work stress → Depression	0.247	0.009	0.144	0.330
Educational Degree^b^	Work stress → Sleep quality	0.389	0.007	0.257	0.542
	Sleep quality → Depression	0.788	0.006	0.718	0.858
	Work stress → Job satisfaction	−0.392	0.009	−0.497	−0.250
	Job satisfaction → Depression	0.006	0.817	−0.076	0.104
	Work stress → Depression	0.099	0.048	0.003	0.223

CI denotes to confidence interval, LB denotes to lower bound, UB denotes to upper bound.

a Denotes to technical secondary school or below.

b Denotes to college degree or above.

**Table 4:** Goodness-of-fit statistics for the multiple group analysis based academic qualifications difference in model 1 and fit indices of model 2.

Models		*p*	CMIN/DF	GFI	AGFI	IFI	TLI	CFI	RMSEA	SRMR	AIC
Model 1	Total	<0.001	3.861	0.911	0.886	0.943	0.933	0.942	0.058	0.0545	1039.144
Model 2	Unconstrained	<0.001	2.596	0.887	0.857	0.937	0.926	0.937	0.044	0.0557	1481.322
	Measurement weights	<0.001	2.557	0.885	0.86	0.936	0.928	0.936	0.043	0.0564	1473.897
	Structural weights	<0.001	2.567	0.883	0.859	0.935	0.927	0.934	0.043	0.0588	1481.612
	Structural covariances	<0.001	2.566	0.883	0.86	0.935	0.927	0.934	0.043	0.0606	1481.788
	Structural residuals	<0.001	2.572	0.882	0.859	0.934	0.927	0.934	0.043	0.0603	1486.622
	Measurement residuals	<0.001	2.678	0.872	0.856	0.924	0.922	0.924	0.045	0.0642	1563.304

CMIN denotes to chi square, DF denotes to degree of freedom.

**Figure 2:**
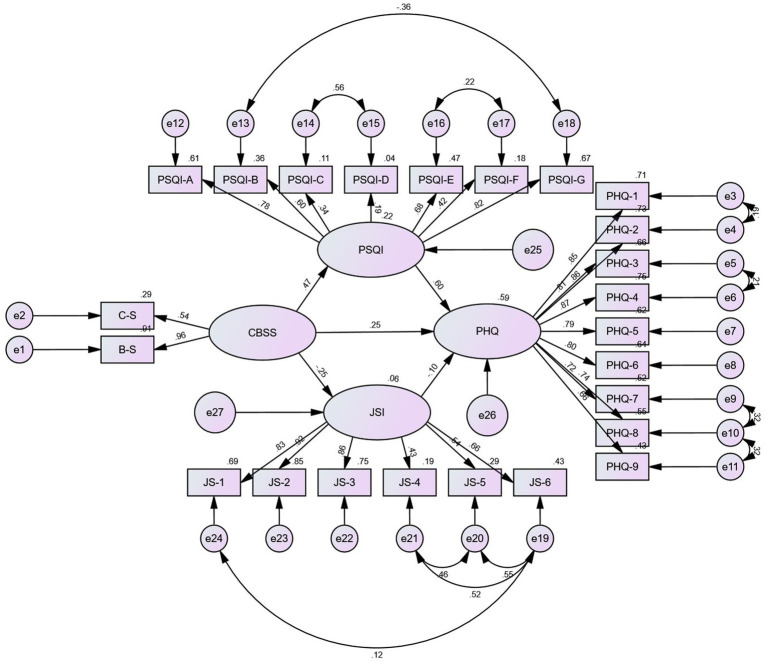
Results of the SEM analysis of the effects of sleep quality and job satisfaction mediate the association between work stress and depression among the sample of technical secondary school and below. All the coefficients in the figure are standardized and significant at 0.001 level. Except that the standardized coefficient of JSI to PHQ is significant at the level of 0.05.

**Figure 3:**
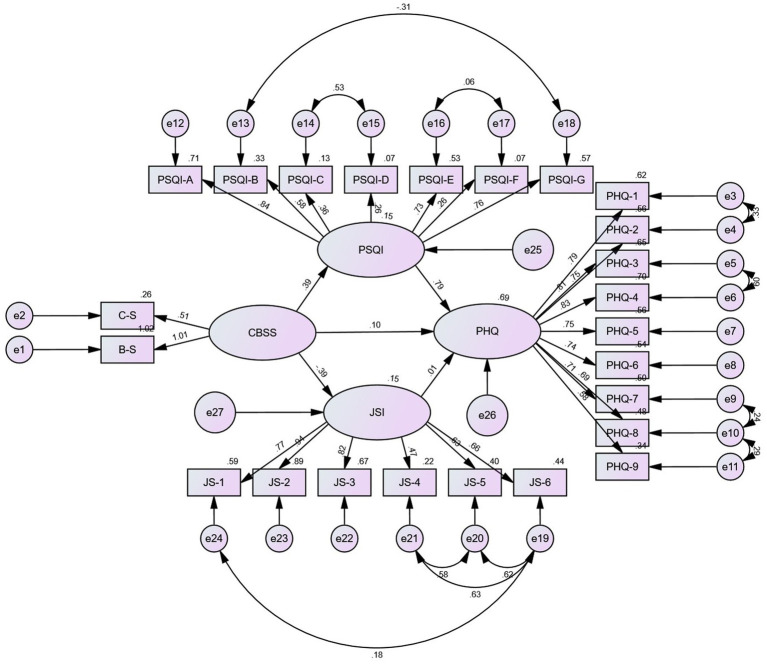
Results of the SEM analysis of the effects of sleep quality and job satisfaction mediate the association between work stress and depression among the sample of college degree or above. All the coefficients in the figure are standardized. The standardized coefficients of CBSS to JSI, CBSS to PSQI and PSQI to PHQ are significant at 0.001 level, CBSS to PHQ is significant at 0.05 level, and JSI to PHQ is not significant.

Work stress (*B* = 0.247, *p* = 0.009), sleep quality (*B* = 0.598, *p* = 0.007), and job satisfaction (*B* = −0.097, *p* = 0.022) all exhibited significant impacts on depression in model 2 and when respondents’ educational levels were technical secondary school or below. Additionally, work stress had statistically significant negative impacts on both job satisfaction (*B* = −0.251, *p* = 0.009) and sleep quality (*B* = 0.465, *p* = 0.010; The PSQI scale score was significantly positively impacted by the Challenge-blocking stress scale score). The relationship between work stress and depression can be mediated by both sleep quality and job satisfaction. The mediating effect between the relationships between the effects of sleep quality on them was 50.6%, while the mediating effect between the relationships in which job satisfaction affected them was 4.4%. In Model 2 and when respondents had a college degree or above, work stress (*B* = 0.099, *p* = 0.048) and sleep quality (*B* = 0.788, *p* = 0.006) significantly affected depression. What’s more, sleep quality (*B* = 0.389, *p* = 0.007) and job satisfaction (*B* = −0.392, *p* = 0.009) were affected by work stress. Job satisfaction was not significant for depression (*B* = 0.006, *p* = 0.817). Sleep quality may be utilized to mediate the association between work stress and depression, whereas job satisfaction cannot. The mediating effect between the relationships between the effects of sleep quality on them was 75.4%. Table 3 shows the details of the results and 95% confidence intervals of the relationships among the variables after the grouping of educational degree. The data analysis results presented in Table 4 indicates that the grouping intermediary model had a good fitting degree. The findings of the multiple group analysis demonstrated that the model with “restricted measurement weights” and that with “restricted structural weights” had significantly different goodness-of-fit statistics (see Table 4). This finding revealed that among HCWs with various educational backgrounds, there were differences in the correlations between work stress, depression, sleep quality, and job satisfaction. Figures 2, 3 show the precise findings of these correlations amongst HCWs with various academic backgrounds. Among them, Figure 2 shows the results of model diagrams with technical secondary school education and below, and Figure 3 reveals the results of model diagrams with college degree or above.

## 4. Discussion

The work stress, depression, sleep quality, and job satisfaction of HCWs were assessed using a sample of 844 HCWs and relevant questionnaires. More importantly, the aim of this study was to investigate the relationship between work stress and depression in HCWs, to further explore the mediating roles of sleep quality and job satisfaction in this relationship, and to compare whether there are variations in the patterns of mediation among HCWs with various educational backgrounds. The findings substantially confirmed the hypotheses overall. This research revealed that sleep quality may moderate a favorable relationship between job stress and depression in HCWs. For HCWs with technical secondary school education and below, job satisfaction can moderate the positive connection between work stress and depression, but this mediation effect was not significant among HCWs with college degrees and above.

It is not new for health care workers that work stress can affect depression levels. Studies have demonstrated their relationship (16, 48). This significant link is undeniable, even within other groups (49). Pressure in the working environment, frequent changes of workplace, time-oppressive work, and irregular schedules such as shifts and staying up late are the major causes of high work pressure for HCWs. These negative factors markedly raise the possibility of occupational depression in HCWs. In addition, work stress may pose an even higher risk for depression by eroding their sense of personal control and feelings of self-worth (50, 51). According to this study, this effect was more pronounced among HCWs with less education than those with more education (0.247 vs. 0.099). However, depression was worse among highly educated HCWs (5.70, SD = 4.63 vs. 4.96, SD = 4.79; *p* < 0.05). Work stress with different educational qualifications is not significant. Greater educated HCWs may be better equipped to mitigate the negative impacts of work stress on depression because of their longer education, higher level of professional expertise, and ability to do so, but they also have a higher chance of developing depression due to other causes. One fashionable view, for instance, is that depression is a “genius disease.” According to studies, more than 75% of poets and 50% of painters experience depression (52). Also, a survey of PhDs discovered that 45% of them had experienced depression (53). Understanding this issue could be made easier by the “cap” impact that education has on mental health. In other words, a mismatch between a high level of education and the demands of the workplace will result in psychological stress, disappointment, and despair, which in turn raises the risk of depressive episodes (54).

This study demonstrated a positive effect between work stress and sleep quality, which was consistent with the existing findings (28, 55). Researchers continue to identify putative mechanisms by which stress affects sleep, even though the mechanism by which job stress causes insomnia is unclear (56). The strain of working night shifts and long hours may contribute to sleep problems, which can exacerbate a number of physical and mental health disorders and diminish one’s productivity (28). Sleep disturbance is the core influencing factor of depression and is also the core symptom. Insomnia in people who are not depressed has been linked in epidemiological studies to a later risk of developing depression (57). A controlled, double-blind study complemented the evidence that treating insomnia reduced the severity of depression and accelerated recovery (23). The aforementioned information may also assist in comprehending how sleep quality influences the link between depression and work stress. This study discovered that the educational background of HCWs had no bearing on the mediating connection; in other words, the mediating role has always persisted regardless of educational background.

The association between work stress and job satisfaction has been the topic of theoretical proposals, empirical investigations, and meta-analyses. This study established a substantial negative correlation between job stress and job satisfaction among HCWs. Specifically, higher work stress was associated with lower satisfaction levels. This was consistent with the relatively new view of many scholars in other populations (58–60). This relationship has also been demonstrated in the HCWs (8). However, few articles have examined the mediating role of job satisfaction between job stress and depression, let alone explored this mediating role in HCWs. In this study, the mediating role of job satisfaction was examined. Job satisfaction was observed to moderate a positive link between work stress and depression among medical practitioners with technical secondary school education or less, but this mediating correlation was not detected in those with college degrees or higher education. Lower educated healthcare professionals’ job satisfaction was substantially correlated with depressive symptoms; highly educated individuals did not confirm this correlation. The reason may be that job satisfaction plays an important role in a group of low educational qualifications as a determinant of personal well-being (61). Increased personal wellbeing and low depression are both results of high job satisfaction. For those with greater levels of education, work satisfaction may no longer play the most crucial role in determining their level of personal wellbeing. Higher education often translates into more and deeper professional knowledge, which makes medical professionals with higher education more self-demanding and consequently more knowledgeable about the complex causes of depression. As a result, work satisfaction is not a good indicator of depression.

Altogether, work stress was strongly associated with depression. Among HCWs with technical secondary school education and below, sleep quality and job satisfaction could mediate a positive relationship between work stress and depression. Among HCWs with a college degree or above, only sleep quality could be used as a mediating variable in the relationship between work stress and depression. HCWs frequently operate in circumstances that are riskier, more unpredictable, and more complicated than those encountered by other employees, particularly considering the recent deterioration in doctor-patient communication. HCW depression at work is a significant public health issue that needs to be addressed but is still underdiagnosed and undertreated. Effective medication management may lower the incidence of severe depressive episodes or improve the ability of people at risk to handle stress at work. The promotion of workplace mental health has been considered as one method of promoting economic growth and maintaining the sustainability of overburdened social welfare systems. Primary prevention may be a viable technique to promote mental health in the workplace. To lessen the prevalence of depression in the workplace, basic preventative strategies as well as high-quality therapies provided by primary care, occupational health, and mental health specialists can be implemented. Regardless of the educational background of the HCWs, lowering work stress can encourage better sleep, which reduces the probability of depression. Policies and actions to lessen the stress on HCWs and enhance sleep quality should be on the agenda of governments and pertinent institutions. Higher educated HCWs report more severe depression, yet little research or effective treatment exists for their depression. Comparatively to less educated health professionals, job satisfaction is not a reliable indicator of depression, thus it’s critical to identify other risk factors and take steps to address them. By improving job satisfaction for HCWs with lower academic qualifications, the risk of depression can be decreased while also enhancing their sense of success and belonging to the institution. In the meanwhile, investigating other aspects in their depression development is equally important and ought to be on the agenda.

There are several limitations to this study worth considering. First, the cross-sectional study could not verify the causal relationship between the variables, nor could it assess the development trend of depression in healthcare workers. Future studies can follow up on a fixed sample of respondents to form longitudinal data to verify the relationship more rigorously between variable cause and effect. Second, the results of this study were derived from only one city, which may limit the generalization of the results among the wider HCWs. Subsequent studies can verify the results of this study by expanding the random sampling range. Third, the content of this survey was self-reported by the target population, and it is difficult to avoid the existence of recall bias.

## 5. Conclusion

The main contributions of this study are: (1) the establishment of a mediating model for the relationship among work stress, sleep quality, and depression among Chinese HCWs; and (2) the discovery that job satisfaction, in groups from technical secondary school and below, mediates the association between work stress and depression. However, this mediating role is not found in groups from colleges and above. The government and relevant ministries should pay attention to and improve the workplace in order to reduce the workload of HCWs, promote sleep quality, and diminish the chance of depression. At the same time, they should increase the job satisfaction of low-educated staff, research the variables influencing depression in highly educated medical staff, and implement proactive and successful depressive disorder prevention strategies.

## Data availability statement

The original contributions presented in the study are included in the article/supplementary material, further inquiries can be directed to the corresponding author.

## Ethics statement

The studies involving human participants were reviewed and approved by Ethical Committee of the Centre for Health Management and Policy Research, Shandong University (approval number: LL20191220). The patients/participants provided their written informed consent to participate in this study.

## Author contributions

All the authors participated in this research. Data analysis and manuscript writing were all completed by AQ. Data was collected by FH, WQ, YD, and ML. The revision of the manuscript was done by AQ, FH, and LX. All authors contributed to the article and approved the submitted version.

## Funding

This research was supported by the National Natural Science Foundation of China (71974118 and 72204145).

## Conflict of interest

The authors declare that the research was conducted in the absence of any commercial or financial relationships that could be construed as a potential conflict of interest.

## Publisher’s note

All claims expressed in this article are solely those of the authors and do not necessarily represent those of their affiliated organizations, or those of the publisher, the editors and the reviewers. Any product that may be evaluated in this article, or claim that may be made by its manufacturer, is not guaranteed or endorsed by the publisher.
